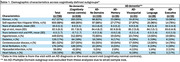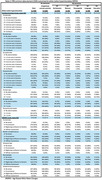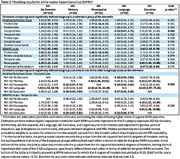# Cognitively‐defined AD dementia subgroups have different MRI white matter hyperintensity distribution in a community‐based cohort

**DOI:** 10.1002/alz70857_107639

**Published:** 2025-12-26

**Authors:** Patti Curl, Rod L Walker, Christine L Mac Donald, Jalal B Andre, Linda K. McEvoy, Kelly Ehrlich, Paul K Crane

**Affiliations:** ^1^ University of Washington Department of Radiology, Seattle, WA, USA; ^2^ Kaiser Permanente Washington Health Research Institute, Seattle, WA, USA; ^3^ University of Washington, Seattle, WA, USA; ^4^ Department of General Internal Medicine, University of Washington School of Medicine, Seattle, WA, USA

## Abstract

**Background:**

Alzheimer's disease (AD) dementia may be heterogenous. Subgrouping by differing cognitive performance has previously correlated with differential brain volume and neuropathology.

**Method:**

Adult Changes in Thought (ACT) is a prospective cohort study, enrolling dementia‐free individuals ^3^age 65 and following them with biennial cognitive testing. ACT participants diagnosed with AD (ACT AD) were divided into subgroups with greater deficits in memory (AD‐Memory), executive function (AD‐Executive), language (AD‐Language), or visuospatial (AD‐Visuospatial) scores on an extensive battery of cognitive tests. ACT AD participants with available proximate MRI were included, as well as 516 no‐dementia controls. Neuroradiologists rated each patient's MRI using NIH neuroimaging common data elements (CDE). Ordinal logistic regression models estimated odds of higher levels of white matter hyperintensities (WMH) associated with each group, adjusted for gender, education, age, and years between diagnosis and MRI. Models incorporated inverse probability weights to account for MRI availability.

**Result:**

MRI within 4 years of diagnosis were available for 220 ACT AD participants (35 AD‐Memory, 31 AD‐Visuospatial, 26 AD‐Language, 18 AD‐Executive, and 110 AD‐no specific subdomain). Demographic and clinical characteristics are presented in Table 1 and WMH findings in Table 2. Model results in Table 3 first show comparisons of the AD‐subgroups to non‐dementia controls (*p*‐value for 5‐degree of freedom (df) joint test of any difference), and then to each other, with the latter limited to WMH outcomes with some evidence of differences between AD‐subgroups themselves rather than just relative to controls (*p* <0.10 for 4‐df test). Estimates suggest: AD‐Visuospatial had more WMH in the occipital lobe relative to AD‐Memory and AD‐Language had more frontal lobe WMH than non‐dementia controls; AD‐Executive had more occipital and temporal lobe WMH than AD‐Memory or AD‐Language, and more infratentorial/cerebellum and basal ganglia WMH than non‐dementia controls.

**Conclusion:**

Increased occipital WMH in AD‐Visuospatial may suggest white matter damage is part of the mechanism of visuospatial impairment. AD‐Executive estimates demonstrated increased WMH in most areas though only achieved significance in some listed above. The relatively low WMH in the temporal lobe demonstrated in both AD‐Memory and AD‐Language may suggest white matter damage is a less important part of the mechanism of impairment.